# Clinical and economic implications of early discharge following posterior spinal fusion for adolescent idiopathic scoliosis

**DOI:** 10.1007/s11832-014-0587-y

**Published:** 2014-04-27

**Authors:** Nicholas D. Fletcher, Nader Shourbaji, Phillip M. Mitchell, Timothy S. Oswald, Dennis P. Devito, Robert W. Bruce

**Affiliations:** 1 Emory University Department of Orthopaedics, 59 Executive Park South NE, Atlanta, GA 30329 USA; 2 Pediatric Orthopaedic Associates, 6 Executive Park Drive, Suite 10, Atlanta, GA 30329 USA; 3 Children’s Orthopaedic of Atlanta, 5445 Meridian Mark Rd, Atlanta, GA 30342 USA

**Keywords:** Adolescent idiopathic scoliosis, Accelerated discharge, Cost of treatment, Posterior spinal fusion

## Abstract

**Objective:**

To evaluate the clinical and economic impact of a novel postoperative pathway following posterior spinal fusion (PSF) in patients with adolescent idiopathic scoliosis (AIS).

**Methods:**

Patient charts were reviewed for demographic data and to determine length of surgery, implant density, use of osteotomies, estimated blood loss, American Society of Anesthesiologists (ASA) score, length of hospital stay, and any subsequent complications. Hospital charges were divided by charge code to evaluate potential savings.

**Results:**

Two hundred and seventy-nine of 365 patients (76.4 %) treated with PSF carried a diagnosis of AIS and had completed 6 months of clinical and radiologic follow-up, a period of time deemed adequate to assess early complications. There was no difference between groups in age at surgery, sex, number of levels fused, or length of follow-up. Patients managed under the accelerated discharge (AD) pathway averaged 1.36 (31.7 %) fewer days of inpatient stay. Operative time was associated with a shorter length of stay. There was no difference in complications between groups. Hospital charges for room and board were significantly less in the AD group ($1.885 vs. $2,779, *p* < 0.001).

**Conclusions:**

A pathway aimed to expedite discharge following PSF for AIS decreased hospital stay by nearly one-third without any increase in early complication rate. A small but significant decrease in hospital charges was seen following early discharge. Early discharge following PSF for AIS may be achieved without increased risk of complications, while providing a small cost savings.

**Electronic supplementary material:**

The online version of this article (doi:10.1007/s11832-014-0587-y) contains supplementary material, which is available to authorized users.

## Introduction

Adolescent idiopathic scoliosis (AIS) is the most common spinal condition requiring surgical treatment during the teenage years. Patients with AIS tend to be otherwise healthy with few, if any, comorbidities. Hospital stay following spinal instrumentation surgery has decreased significantly since its inception, when patients were hospitalized for 3 weeks or more [1]. Despite technological improvements, hospital stay continues to average between 4.2 and 9.3 days following surgery in North America [2–9]. Shortening the length of hospital stay using a “fast-track” pathway following major orthopaedic procedures that permit early mobilization has been demonstrated to be safe and efficacious in patients undergoing joint arthroplasty [10–13]. There are no published studies documenting similar pathways in pediatric spinal surgery.

The purpose of this study was to evaluate the clinical and economic implications of an accelerated discharge (AD) pathway when compared to a more standard discharge pathway (SD). We hypothesized that the AD pathway would lead to equivalent complication rates with a reduction in the overall cost associated with posterior spinal fusion (PSF) in patients with AIS.

## Methods

Institutional review board approval was obtained prior to initiation of this study. A retrospective review of patients who underwent PSF for AIS treated at two hospital campuses within the same hospital system between 2006 and 2008 was performed. Patients treated for AIS with PSF were identified by a query of hospital billing records based on ICD-9 codes for AIS and CPT codes related to PSF. One hospital instituted an AD pathway (see Appendix in the electronic supplementary material), while the other maintained the SD pathway (see Table 1). The AD pathway was developed so as to emphasize early mobilization. Surgical drains and Foley catheters were removed the morning after surgery and patients were started on a regular diet. A patient-controlled anesthesia (PCA) pump was used for immediate pain control, but was discontinued the morning after surgery and transition to oral narcotics and antispasmodics (diazepam). Patients were given IV Ketorolac on a case-by-case basis based on surgeon preference. Physical therapy was started on postoperative day #1, and patients were seen twice a day for physical therapy, with families encouraged to mobilize the patients as much as tolerated. Nurses and therapists were educated on the postoperative pathway and a formal electronic order set was made to ensure compliance. Patients were discharged home following their hospital stay regardless of whether they had a bowel movement. The SD pathway was somewhat less regimented and followed a more traditional hospital course encountered by postoperative spinal fusion patients. Drains and Foley catheters were left until the patient was mobilized. A regular diet was started only after patients were passing flatus and the PCA was typically left on for 2 days postoperatively. More variability in the SD group was related to the fact that pathway was a surgeon-directed pathway, rather than the truly coordinated multidisciplinary pathway seen in the AD patients.

**Table 1:** Accelerated versus standardized pathways

Postoperative unit	Surgical floor	
	Standardized discharge	Accelerated discharge
Transition to oral pain meds from PCA pump	POD#2	POD#1
Foley removed	POD#1–2	POD#1
Drain removed	POD#2–3	POD#1–2
Mobilize with PT	Minimum of once daily begin POD#1	2× daily begin POD#1
Transition to solid diet	Surgeon’s discretion	POD#1 as tolerated
Discharge	POD#4	POD#2–3

*POD* postoperative day, *PCA* patient-controlled anesthesia

Patients between the ages of 10 and 18 years met the inclusion criteria if they underwent PSF for AIS and had 6 months of documented follow-up. Complications after 6 months were deemed unlikely to be related specifically to the length of stay or postoperative care pathway. Patients treated with anterior or combined anterior/posterior spinal fusion, those treated for congenital, syndromic or neuromuscular scoliosis, those treated with growing spine instrumentation and those with inadequate follow-up were excluded. One surgeon at the AD hospital declined to adhere to the AD pathway, so his patients were excluded.

Patient charts were reviewed for demographic data and to determine length of surgery, number of fusion levels, implant density, American Society of Anesthesiologists (ASA) score as determined by the anesthesiologist, estimated blood loss (EBL), length of hospital stay, and any subsequent complications. Duration of surgery was calculated based on anesthesia records from the time of incision to the time of wound closure. Hospital stay was calculated from the time the patient left the operating suite to the time at which the nursing staff formally discharged the patient from the hospital floor. Complications were determined based on a review of both clinic and hospital records.

Complications following PSF were divided into five categories based on severity and etiology. Category 1 was designated for medical complications resulting in an atypical or prolonged postoperative course or readmission in the perioperative period. These included pulmonary compromise, gastrointestinal difficulties other than a typical delay in postoperative bowel movements, urinary infection, incontinence unrelated to neurological injury, vascular, or neurologic other than spinal cord injury. Category 2 included wound complications following fusion that were treated expectantly, including mild wound dehiscence without drainage. Category 3 covered those wound complications related to the fusion necessitating treatment with antibiotics or local wound care but no surgical intervention. Category 4 included those patients who developed a wound infection following fusion requiring surgical debridement or drainage. Category 5 included patients who required repeat operation within the first 6 months postoperatively for non-infectious causes.

Hospital charges were reviewed for all patients and divided by charge code as obtained from the hospital finance department. A direct cost comparison is afforded by the fact that both campuses are in the same billing system. Each chargeable item is given a charge description master (CDM) code within the hospital billing system. These CDM codes can then be categorized based on detailed revenue codes such that all charges can be separated.

### Statistical analysis

Chi-squared analysis was used to compare rates between hospitals for the categorical variables in this study, and a two-sample *t* test was used to compare the mean values of the continuous variables for the two hospitals. When comparing complication rates, the Fisher exact test was used, as the number of complications was too small to use the Chi-squared test. The Spearman correlation coefficient was used to measure the dependence of length of stay on ASA score, EBL, number of implants used, and length of surgery. The Spearman rho correlation coefficient was used because the value of the correlation was much less vulnerable to the effect of outliers. Analysis of covariance was used to compare the length of stay for the hospitals, when adjusted for time in surgery.

## Results

Four hundred and eighty-six patients underwent spinal fusion from 2006 to 2008 at our combined center. Of these patients, 122 carried diagnoses other than AIS. Of the 364 patients treated with spinal fusion for AIS, 316 (86.8 %) underwent posterior spinal fusion with segmental instrumentation and completed follow-up. Thirty-seven patients were excluded who underwent PSF at the AD campus and were not placed on the AD protocol based on surgeon preference and noncompliance with the pathway. This left a total of 279 (76.4 %) patients for analysis. One hundred and fifty-four patients were treated with the AD pathway and 125 with the SD pathway. Patients cared for at the AD campus underwent PSF by one of two staff surgeons and were managed using the AD pathway, which was directed by a multidisciplinary team. Patients cared for at the SD campus underwent PSF by one of six staff surgeons and were cared for using a surgeon-directed pathway. No surgeon operating at the AD hospital also cared for patients at the SD hospital and vice versa. All surgeons are fellowship trained pediatric orthopaedic surgeons of varying levels of seniority.

### Clinical analysis

There was no difference at the time of surgery with regards to patient age, gender, number of levels fused, and length of follow-up between the AD and SD groups (see Table 2). Patients in the AD pathway were more likely to carry a higher ASA score of 2 or 3 (38.3 vs. 33.2 %, *p* = 0.03). One hundred and fifty-four patients underwent PSF at the AD campus and 125 at the SD campus. The surgical time was significantly shorter (220 ± 45 vs. 312 ± 68 min, *p* < 0.0001) and EBL significantly less (336 ± 313 ml vs. 763 ± 556, *p* < 0.0001) in the AD group, although there was no standardized measurement of EBL between hospitals and it was impossible to determine whether the treating surgeon waited for clinical evidence of intact neurological function prior to leaving the operating room. Patients in the SD pathway were instrumented with more implants (17.4 ± 3.4 vs. 15.2 ± 3.2, *p* < 0.0001), including a greater number of pedicle screws (15.7 ± 4.6 vs. 13.7 ± 3.5, *p* = 0.0001), resulting in a higher implant density defined as the number of implants per level fused (1.67 ± 0.3 vs. 1.54 ± 0.3, *p* < 0.0001), and were more likely to have Ponte osteotomies (5.2 vs. 30.1 %, *p* = 0.03) than those in the AD pathway. 61 % of patients at the SD campus had robotically or navigationally assisted screw placement compared to zero at the AD campus.

**Table 2:** Comparison between hospitals using the accelerated discharge (AD) and standard discharge (SD) pathways

	AD hospital	SD hospital	*p* value
Age	14.4 ± 1.9	14.7 ± 2.3	0.22
Female sex	85.7 %	80.1 %	0.21
ASA > 1	38.3 %	33.2 %	0.03
Number of levels fused	10.1 ± 2.09	10.5 ± 1.82	0.10
Number of implants (excluding rods)	15.2 ± 3.2	17.4 ± 3.4	<0.0001
Implant density (implants per level fused)	1.54 ± 0.3	1.67 ± 0.2	<0.0001
Number of pedicle screws	13.76 ± 3.5	15.65 ± 4.6	0.0001
Ponte osteotomies	5.2 %	30.1 %	0.04
Use of navigation/robotics	0 %	61 %	<0.0001
Length of surgery (min)	220 ± 45	312 ± 68	<0.0001
Estimated blood loss (ml)	336 ± 313	763 ± 556	<0.0001
Length of stay (days)	2.92 ± 0.71	4.28 ± 1.08	<0.0001
Length of follow-up (days)	772 ± 406	847 ± 455	0.15

Patients treated using the AD pathway were discharged an average of 2.92 ± 0.71 days postoperatively compared to 4.28 ± 1.08 days under the SD pathway, a difference of 1.36 days or 31.7 % (*p* < 0.0001). Three patients (1.9 %) treated at the AD hospital spent time in the intensive care unit (ICU) compared with nine patients (7.1 %) at the SD hospital (*p* = 0.01). The Spearman correlation showed a modest correlation between length of stay and both EBL (*ρ* = 0.43, *p* < 0.0001) and length of surgery (*ρ* = 0.57, *p* < 0.0001) when considering all patients. However, the Spearman correlation between length of stay and EBL was reduced to a non-significant value when computed for each individual hospital. Analysis of covariance revealed that the AD pathway reduced hospital stay by 0.99 days after adjusting for the linear effect of time in surgery. The analysis also revealed that each extra hour in the operating room increased the length of stay by 0.24 days, regardless of the postoperative pathway (*p* < 0.05). Implant density, blood loss and ASA score failed to correlate with length of stay in other analyses of covariance.

### Complications

Complications were divided into five classes (Table 3). There was no statistical difference between patients treated in the AD and SD pathways with regards to medical complications, minor wound complications not requiring treatment, wound complications requiring a course of oral antibiotics, or wound complications requiring surgical debridement. Two patients (1.6 %) within the SD group underwent reoperations within the 6 months following surgery for non-wound related complications. No patients within the AD group had a second operation for non-wound related causes. Due to the very low rate of complications following spinal fusion, a post hoc power analysis revealed that this study was not adequately powered to show a difference in complications between groups.

**Table 3:** Comparison of complication rates by percentage of total surgical procedures between hospitals using the accelerated discharge (AD) and traditional discharge (TD) pathways

Complication	AD hospital (%)	SD hospital (%)	*p* value
Non-wound: medical	2.60	1.60	1.00
Wound: no treatment	1.30	0	0.51
Wound: nonsurgical	8.44	4.80	0.25
Wound: surgical	3.25	2.40	1.0
Revision surgery: non-wound	0	1.6	0.55

See text for description of complication type. There was no significant difference between total complications rates between hospitals (*p* = 0.17)

### Economic impact

Use of the AD pathway resulted in a 33 % decrease in room charges ($1,885 ± 486 vs. $2,779 ± 617, *p* < 0.0001) and an 11 % decrease in therapy charges ($554 ± 201 vs. $619 ± 219, *p* = 0.004). Surgeons did not utilize the same implant systems or bone graft for their spinal fusion, and contracts between venders were not identical between hospitals. Charges for implants and bone graft were therefore excluded from analysis (Table 4). A post hoc power analysis showed that this study was adequately powered to show a difference between groups with regards to room charges.

**Table 4:** Hospital charges for services provided

	Accelerated discharge	Standard discharge	*p* value
Laboratory	$2,212 ± 679	$2,212 ± 903	0.99
PACU	$743 ± 43	$757 ± 50	0.08
Physical therapy	$554 ± 201	$619 ± 219	0.004
Radiology	$260 ± 106	$260 ± 87	0.01
Room and board	$1,885 ± 486	$2,779 ± 617	0.0001

Operating room implant charges could not be compared given differences in vendors

## Discussion

Recognizing a need to reduce postoperative care costs while optimizing outcomes following PSF for AIS, a task force within our hospital system was organized to develop a novel postoperative care pathway. Surgeons within our hospital system operate at one of two campuses, and thus a multidisciplinary team consisting of nursing, physiotherapy, pharmacy, respiratory therapy, anesthesia, and scoliosis surgeons from both campuses was convened and a new care pathway (SD) defined with the initial goal of patient discharge on postoperative day 4. At one campus, the pathway was implemented in a systematic fashion. There was widespread nursing education to minimize variability in patient care and establish expectations for an early discharge with patient families in the perioperative period. This streamlined approach quickly resulted in an AD on the second or third day postoperatively, and the pathway was updated to reflect these improvements. At the second campus, patients were managed under the SD pathway, and discharge was anticipated on the fourth or fifth day postoperatively.

The AD pathway resulted in a significantly shorter hospital stay when compared to patients managed by the SD pathway. The postoperative stay of 2.92 days is 31.7 % shorter than that of patients in the SD pathway and between 33 and 65 % shorter than published averages in the North American literature [2–8]. This decrease in hospital stay has many potential benefits, including lessening the potential exposure to nosocomial infection [14–18], a quicker transition to the home setting for patients and for parents with multiple children, and earlier return to work for the parents. Given the retrospective nature of this study, it is difficult to say what aspect of the pathway provides the biggest impact with regards to early discharge; however, the establishment of an expectation for discharge on the second hospital day is crucial to the pathway’s success [19–24].

Our hospital system includes two hospital campuses; however, the surgeons occupying them represent three different clinical practices. As such, pathway developments or changes are not mandated across all groups. The original care pathway was developed with collaboration between both campuses; however, the further acceleration of discharge at one hospital was successful due in large part to the staff surgeons at that campus championing the pathway and educating the nursing staff and therapists. The ability to shorten the hospital stay by minimizing postoperative variability in care highlights the benefits of a coordinated team approach to scoliosis care.

One reason that early discharge following spinal fusion has not been previously adopted may be a concern for a higher complication rate and rehospitalization. Early complications are often related to spinal instrumentation itself (i.e., malpositioned screw), wound healing or infection, and medical issues such as ileus or respiratory distress. When accounting for complications other than those associated with instrumentation itself, we found no difference in either medical or wound related complications between patients treated using the AD and SD pathways. In particular, there was no significant difference between groups with regards to wound or medical complications. Our outcomes suggest that many of the postoperative tendencies following PSF for AIS may be based in dogma, such as waiting for the return of bowel function and tolerance of a regular diet, or the need for prolonged IV narcotic analgesia.

The adoption of the AD pathway provided true cost savings, as charges for room and board were decreased 33 % compared to those treated in the SD pathway, with a small decrease in therapy charges. These charges are less than those described in previous studies, although this variability may be related to both geographic and demographic differences [2, 25, 26]. While this savings is significant, it remains a much smaller component of the overall health care cost incurred by the patient or insurance provider in North America as the price of surgical implants continues to far surpass that of hospital stay or ancillary services [2]. The $900 saved in room charges in our system is but a small fraction of the total hospital charge in the United States; however, this savings may be much more substantial when imparted in other countries where implant costs are lower and the cost of hospital stay is higher. We were unable to truly compare the savings imparted by the AD pathway, as the variability in implants was significant. Nonetheless, the savings imparted by utilization of this pathway is likely comparable to using one fewer pedicle screw [27]. It should be noted, however, that when a savings of between 33 and 65 % is applied across multiple surgical procedures and over extended periods of time, financial impact could be quite significant as long as complications are not increased. Another system-based consideration is the potential cost savings associated with freeing up hospital beds to accommodate other patients and obviate the need to expand hospital facilities. When extrapolating the data for the two hospitals, the implementation of the AD pathway at the SD hospital could have resulted in 170 fewer hospital days for the 125 patients treated at that campus over 2 years.

Differences were noted between groups with regards to EBL, length of surgery, and implant density. Regression analysis failed to show correlation between implant density, EBL and length of stay. Of patients treated at the hospital using the SD pathway during this period, 61.1 % were treated during the early experience with navigationally directed spinal instrumentation. It is difficult to ascertain the exact amount of time added by the use of navigation, given the retrospective nature of this study [28, 29]. More patients at the SD campus were treated with Ponte osteotomies, which also add to surgical time and blood loss [30]. Preoperative curve magnitude could not be determined for many patients due to inadequate preoperative radiographs obtained at satellite clinics prior to implementation of an electronic PACS system. Larger curves and higher implant densities have been noted to impact surgical time without increasing complications and may have affected the length of surgery [8, 31, 32]. We attempted to account for this variability by evaluating surgical levels fused, which was not different between campuses.

This study has several limitations, including a lack of validated outcomes with regards to patient and family contentment with the experience. Initial concerns about patient pain control and ability to transition to the home setting early in the postoperative period have been largely unfounded in our experience, and the AD pathway has been adopted across our entire hospital system since 2009. A second limitation may be that the retrospective nature of this study prevented our ability to capture all complications. Our hospital system provides the majority of scoliosis care to the state; however, it is possible that a patient may have gone to a more regionally accessible hospital to have a complication addressed. We assumed that early postoperative issues would have likely been relayed to the treating surgeon. Also, some wound-related complications may have been addressed by ancillary staff members without documentation in the hospital chart. A third limitation was our inability to evaluate preoperative radiographs, as one of the practices did not keep films from this time period. We attempted to compensate for this lack of imaging by instead evaluating levels fused. Finally, while the AD and SD pathways were implemented by the surgeons at each campus, there is no way to ensure complete compliance, given the retrospective nature of this study.

In conclusion, a system-based and pathway-driven pathway was created, and successfully decreased hospital stay following PSF for AIS without an increase in complications. A small, but real, cost savings was realized through use of this pathway. Hospitals that perform a large number of PSF procedures should consider adopting a similar pathway to minimize variability and maximize efficiency without compromising patient outcome. Further study is required to evaluate the patient experience using an AD pathway.

## Electronic supplementary material

Below is the link to the electronic supplementary material.

Supplementary material 1 (PDF 360 kb)

Supplementary material 2 (PDF 392 kb)
