# Supplementary material for: Clinical and economic implications of early discharge following posterior spinal fusion for adolescent idiopathic scoliosis
Source: J Child Orthop. 2014 Apr 27;8(3):257–63. doi: 10.1007/s11832-014-0587-y (PMC4142881; doi:10.1007/s11832-014-0587-y)
Supplement: Supplementary file 1 — Supplementary material 1 (PDF 360 kb) [file 11832_2014_587_MOESM1_ESM.pdf]

Spinal Fusion Surgical Pathway:
Prior to Surgery, Pre-op, Intraop, PACU (phase 1)

Updated 6/11/2013

This pathway is a general guideline and does not represent a professional care standard governing providers' obligation to patients. Ultimately the patient's physician must determine the most appropriate care.

Timeline	Pre-op Visit	Day Surgery	Day of Surgery	Day of Surgery
Unit	**Pre-op Clinic**<br>**(seen ≤ 14 days before surgery)**	**Day Surgery**	**Operating Room**	**PACU**
Assessment & Monitoring	VS<br>Height and Weight<br>Prior to admission medications documented	•Admit to Day Surgery room 1.5 hours prior to scheduled procedure<br>• Height and Weight recorded<br>•Nursing assess per protocol<br>•Physician completes H&P<br>•Physician obtains consent<br>•RN checks consent with Intraop RN when transfered to the OR suite	•Check consent with Day Surgery RN<br>•Assessment per protocol<br>•Utilize Cell Saver<br>•Neuro Monitoring<br>**End of Procedure:**<br>•Counts correct<br>•Procedure performed<br>•Wound class with surgical team	VS with Neurovascular Checks<br>Verify EBL and document in Optime*
Laboratory:	H&H<br>Type and Crossmatch<br>**Day Prior to Surgery:**<br>Ensure blood is available prior to day of surgery	•Ensure H& H results available<br>•Ensure Type & Cross completed<br>•Verify Designated Donor blood if available	**Pre-Procedure:**<br>Verify blood is available<br>**End of procedure:**<br>•Verify EBL*<br>•Document blood products infused<br>•Specimens sent to lab	H & H Stat
Radiology			Ensure PACS is working properly and films are displayed prior to incision	T-L spine if not completed in OR<br>CXR if CVL or Chest Tube
Medications & IV Therapy		Pre-op medications per Anesthesia	Per Anesthesia	Initiate Pain Management plan per admission orders
		**Optional:**<br>Neurontin Loading Dose 15mg/kg with a maximum dose of 1200mg PO with sip of water per Physcian	Antibiotics order to anesthesia per surgeon<br>Infusion guidelines per Infection Prevention guidelines<br>Dual Therapy:<br>•Ceftazidime loading dose:<br>50 mg/kg (max 2 gm)<br>then25 mg/kg (max 1 gm) every 3 hours during surgery (maximum of 3 intraoperative doses)<br>•Vancomycin 20 mg/kg (max 1 gm) preoperative over 1 hour (incision @ 30 minutes) then every 8 hours x 2 doses	
Treatments & Procedures	CHG product sent home	Pre-operative cleansing using CHG by Day Surg/Inpatient staff<br>Perform pre-op skin assessment		Assess skin integrity
Nutrition GI	NPO instructions and arrival times/teaching completed	NPO	NPO	NPO
Activity		Empty bladder prior to surgery		Place on Inpatient bed<br>Bed Rest & Log Roll
Consults	Anesthesia<br>Child Life	Anesthesia<br>Neuro monitoring	Autotranfusionist	
Partnering with Parents & Education	CHG teaching (insert link to teaching sheet)<br>After Anesthesia Care<br>Preparing Your Child for Surgery<br>Spinal Fusion (revised)<br>Spinal Fusion Movement (revised)<br>Pain Management<br>Dental Health	Reinforce education from Pre-op		Reinforce education

***Estimated Blood Loss (EBL) for Spinal Fusion cases:**
- At the end of the surgical case, the Surgeon, Anesthesia and Autotranfusionist agree on fluids infused, estimated blood lost(EBL) and add 20% to allow for immeasurable blood loss.
- Surgeon documents EBL in brief op note.
- The final EBL is the number documented on the anesthesia form. Anesthesia communicates the EBL to the PACU nurse.
- The final EBL is documented in OP time by the PACU nurse and will be labeled EBL on the documentation flow sheet and should be used by the surgeon when dictating the operative note.
