# Supplementary material for: Clinical and economic implications of early discharge following posterior spinal fusion for adolescent idiopathic scoliosis
Source: J Child Orthop. 2014 Apr 27;8(3):257–63. doi: 10.1007/s11832-014-0587-y (PMC4142881; doi:10.1007/s11832-014-0587-y)
Supplement: Supplementary file 2 — Supplementary material 2 (PDF 392 kb) [file 11832_2014_587_MOESM2_ESM.pdf]

Spinal Fusion Surgical Pathway

Phase II - Post-op Management & Discharge

Goal: Discharge on Post-op Day 2/3 **Updated 6/11/2013**

<u>Discharge Criteria:</u>

- Tolerating regular diet
- Pain controlled with oral medications
- Ambulate without assistance per PT Protocol
- Caregivers verbalize spinal fusion precautions and activity modifications

This pathway is a general guideline and does not represent a professional care standard governing providers' obligation to pa tients. Ultimately the patient's physician must determine the most appropriate care.

Timeline	Surgical Day	Post-op Day 1	Post-op Day 2	Post-op Day 3
**Unit**	***Admit to Inpatient*** **: General floor prefered**	Inpatient	Inpatient	Inpatient
**Assessment & Monitoring**	VS q 4hr including Braden Q every 12hr<br>Neurovascular (NV) checks q 2hr<br>Continuous Pulse Ox & O2 to keep sats > 93%<br>Strict Intake & Output q 4hr including drains	VS q 4hr including Braden Q<br>Neurovascular (NV)checks q 4hr<br>Strict Intake & Output q4hr including drains<br>***Wean O2 to keep sats > 93%***	VS q 4hr including Braden Q<br>Neurovascular (NV)checks q 4hr<br>Strict Intake & Outputq 4hr including drains<br>***Discontinue Pulse Ox if sats > 93% on RA and PCA pump discontinued***	VS q 4hr including Braden Q<br>Neurovascular (NV)checks q 4hr<br>Discontinue PO if sats > 93% on RA and PCA pump discontinued<br>***Routine Intake & Output*** including drains
**MD Notification**	**<u>Notify MD:</u>**<br>Change in VS or NV status<br>UOP < 0.5ml/kg/hr times 2hr<br>Hemovac output is >200ml/8 hrs	**<u>Notify MD:</u>**<br>Change in VS or NV status<br>Hemovac output is >200ml/8 hrs<br>***UOP < 1ml/hr/kg over 4hrs***<br>***No void in 6hrs after foley discontinued***	**<u>Notify MD:</u>**<br>Change in VS or NV status<br>UOP < 1ml/hr/kg over 4hrs<br>Hemovac output is >200ml/8 hrs	**<u>Notify MD:</u>**<br>Change in VS or NV status<br>Hemovac output is >200ml/8 hrs
**Laboratory**	H&H in am	H&H @ 0500		
**Radiology**		***Upright T/L Spine x-ray 2 views follow-up if not done in PACU***		
**Medications & IV Therapy**	IV Fluids<br>Zofran IV 0.1 mg/kg per dose (max dose of 4 mg) IV q8h PRN N/V<br>**<u>Antibiotics: Dual Therapy</u>**<br>Vancomycin 20 mg/kg (max 1gm) IV q8 hours times 2 doses<br>Ceftazidime 25mg/kg (maximum dose 1gm) IV q 8 hours times 2 doses	IV fluids - ***INT IV & discontinue IV Fluid*** when tolerating PO liquids without N/V<br>***Discontinue antibiotics after 24hrs***<br>***Start Miralax 0.5 gram/kg everyday, with a max dose of 17 grams***	INT IV if tolerating PO liquids<br>Continue Miralax<br>***Consider MOM or Dulcolax Suppository if bowel sounds present and no stool***	Discontinue IV<br>Continue Miralax<br>Consider MOM or Dulcolax suppository if bowel sounds present and no stool
**Pain Control**	**<u>Pain Control:</u>**<br>Valium 0.1 mg/kg IV q4h PRN muscle spasticity<br>PCA pump with bolus doses<br><br>**<u>Optional:</u>**<br>Neurontin 5mg/kg TID, PO, max 300mg TID<br>Toradol 0.5mg/kg IV q 6hr, max 8 doses<br>- Zantac 1 mg/kg/day IV q 8h (max dose 50mg)if using Toradol<br>ONQ pain pump with Bupivacaine	**<u>Pain Control:</u>**<br>Valium 0.1 mg/kg IV q4h PRN muscle spasticity<br>***Discontinue PCA Pump***<br>***Start <u>Percocet</u> OR <u>Norco</u> PO Routine q 4hr***<br>***IV Morphine Sulfate*** for breakthrough pain<br>***Toradol 0.5mg/kg IV q 6hr, max 8 doses***<br>***- Zantac 1mg/kg/day IV q 8 hr, (max dose is 50mg) if using Toradol***<br>**<u>Optional:</u>**<br>Neurontin 5mg/kg TID<br>ONQ pain pump with Bupivacaine	**<u>Pain Control:</u>**<br>IV Morphine Sulfate for breakthrough pain<br>***<u>Percocet</u> OR <u>Norco</u> q 4hr PRN pain***<br>***Change Valium to PO q 4hr PRN muscle spasticity***	**<u>Pain Control:</u>**<br>Discontinue Toradol<br>Continue pain management program until discharged
**Respiratory & Treatments; Procedures**	Incentive Spirometry q 2hr when awake<br>Check Surgical dressing q 4hr & reinforce PRN<br><br>Foley to staight drain	Incentive Spirometry q 2hr when awake<br>MD to discontinue drains<br><br>***Discontinue foley if UOP >1ml/kg/hr AND PCA is discontinued***	Incentive Spirometry q 2hr when awake<br>MD to discontinue drains	Incentive Spirometry q 2hr when awake
**Nutrition GI**	***Ice chips and sips of clears as tolerated***<br>Assess bowel sounds	***Clears - Advance diet as tolerated***<br>Assess bowel sounds<br>Encourage gum chewing	***Regular diet as tolerated***<br>Encourage gum chewing	Regular Diet as tolerated<br>Encourage gum chewing
**Activity**	Log roll q 2hr and PRN until patient is rolling independently	Log roll q 2hr and PRN until patient is rolling independently<br>OOB to Chair TID<br>Ambulate TID	Continue to log roll independently<br>OOB to Chair TID<br>Ambulate TID<br>Begin stairs	Continue to log roll independently<br>OOB to Chair TID<br>Ambulate TID<br>Conquer stairs
**Consults**	***Case Management*** to assess for Durable Medical Equipment	***PT to see patient 2xday***<br><br>***Child Life***	PT to see patient 2xday<br>***OT to see patient for ADLs x1***<br>Child Life	PT to see patient 1 xday<br><br>Child Life
**Partnering with Parents & Education**	**Reinforce Education** (Teaching Sheets)<br>Pain management<br>Spinal Fusion<br>Spinal Fusion Movement:<br>***Log rolling and sidelie to sit & sit to stand transfers***	Reinforce Education (Teaching Sheets)<br>Spinal Fusion Movement:<br>**Ambulation**<br><br>***Partner with parents for ambulation schedule***	Reinforce Education (Teaching Sheets)<br>Spinal Fusion Movement:<br>***Precautions and body mechanics***<br><br>Partner with parents for ambulation schedule	***Home Care Teaching Sheets***<br><br><br><br>Partner with parents for ambulation schedule
**Discharge Planning**	Assess Home Health Needs<br>Assess Transportation Needs<br>Provide Family with written needs	***Assess Home Health Needs***	Ensure Home Health needs are available<br>***Ensure Transportation needs are available for next day***<br>***Plan for follow-up arranged with Physician***	Ensure Home Health needs are available on discharge<br>***Plan for follow-up arranged with Physician***

Confidential Privileged Quality Peer Review Material Pursuant to GPR Statue Georgia, Code Sections 31-7-130 to 133.

Not Part of the Medical Record
